# Precise Numerical Differentiation of Thermodynamic Functions with Multicomplex Variables

**DOI:** 10.6028/jres.126.033

**Published:** 2021-11-29

**Authors:** Ulrich K. Deiters, Ian H. Bell

**Affiliations:** 1 1 Institute of Physical Chemistry, Faculty of Mathematics and Natural Sciences, University of Cologne, D-50939 Köln, Germany; 2 National Institute of Standards and Technology, Boulder, CO 80305, USA

**Keywords:** multicomplex arithmetics, numerical differentiation

## 1 Introduction

Differentiation of functions plays a major role in many disciplines of science, from physics to engineering. A special case is thermodynamics, which is practically built upon differentiation: thermodynamic theories and models usually represent observable properties of matter as derivatives of some master functions, e.g., the Helmholtz energy or the Gibbs energy. For example, pressure is a derivative of the Helmholtz energy with respect to volume (at constant temperature), or chemical potentials are derivatives of master functions mentioned above with respect to amount of substance. In particular, stability criteria are often related to second-order derivatives of the master functions; for example, mechanical stability depends on the sign of the isothermal compressibility, which in turn is related to a second-order derivative of the Helmholtz energy with respect to volume.

Differentiation usually does not pose a principal problem, as most functions that one encounters in science or engineering are analytic. The calculation of derivatives can, however, pose a practical problem, as functions (with the exception of polynomials) tend to become more complicated upon differentiation and computationally more costly to evaluate. Differentiating the functions of complicated thermodynamic models "by hand" is therefore often neither a pleasant nor an economical task. An alternative are symbolic-algebra computer programs. These can not only perform differentiations of arbitrary complexity, but also?"within limits?"simplify the results. Such programs are, however, rather expensive and not generally available. Moreover, they may not be applicable if the operand function cannot be written down explicitly, for instance because it contains terms that require iterations.

Such equations of state for fluids do indeed exist:

• The equation of state of Dieterici [1] of 1899 was published in a pressure-explicit form; it cannot be integrated analytically. This equation is not obsolete: Sadus [2, 3] reported some favourable thermodynamic features in 2001. Polishuk and Vera predicted critical curves of mixtures, but had to use a truncated version of this equation, which could be integrated analytically.
• Boshkova and Deiters used temperature- and density-dependent hard-core volumes (obtained from perturbation theory by means of an iterative procedure) to extend equations of state to very high pressures [4].
• Equations of state comprising chemical association models almost always contain a variable that represents the fraction of not associated molecules or binding sites. Examples are the SAFT equation of state [5, 6] and the numerous models derived from it.

There exist program libraries for implicit symbolic differentiation, e.g., autodiff for C++ programs [7], but the integration of the code into existing programs is not always easy.

This leaves numerical differentiation methods. The most widely known ones are finite-difference methods like, e.g.,


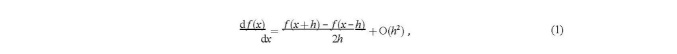


for first-order derivatives. Here *h* is a sufficiently small increment. The proper choice of *h*, however, is not easy: A too small value causes a cancellation of significant digits in the difference, whereas a too large value increases the error due to the O(*h*^2^) term. The latter problem was partially solved by Romberg, whose algorithm makes the *O*(*h*^2^*^n^*) terms cancel by means of a clever superposition of several finite differences [8], and by Ridder, who combined Romberg's method with an iterative scheme that yields the smallest possible error of the derivative [9]. Ridder's method is sufficient for many applications, but certainly not for all, particularly if higher-order derivatives are needed.

An evident remedy is the use of computer arithmetics of more than standard "double" precision. In C++ programs, for instance, this can be accomplished with the boost::multiprecision library. Unfortunately, the use of higher-than-double precision arithmetics significantly slows down the program execution.

Numerical differentiation with almost machine precision, even for higher-order derivatives, is possible with "complex integration" methods. These are based on Cauchy's integral theorem, which relates the path integral along a closed path around a point in the complex plane to derivatives at that point. For derivatives at real-valued locations the method of Lyness and Moler [10] can be recommended, which determines the number of integration nodes automatically while avoiding superfluous invocations of the argument function. A short description is given in Sec. 8. A disadvantage of the method is, however, that it cannot be applied directly to mixed derivatives to functions of more than one variable. This requires either a generalization of the method which involves multi-dimensional integration or the combination of several derivatives through the formalism of directional derivatives (as proposed, for instance, by Deiters and Bell [11, supporting information]).

In this article we want to draw attention to an alternative technique for numerical differentiation that is applicable to pure as well as mixed derivatives of arbitrary order and yields derivatives with almost machine precision, namely the multicomplex step method. This technique is not new, but we feel that it is not as well known as it should be.

## 2 Theory

Before we come to the discussion of the differentiation methods it is necessary to emphasize that we are, in principle, seeking derivatives of real-valued functions for real-valued arguments. Even if the methods that are described in the following sections require a generalization of the argument domain as well as the co-domain to complex (or even multicomplex) numbers, the functions considered here have the property that they return a real value if their arguments are all real,


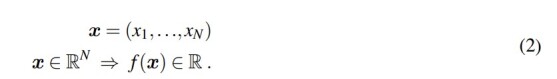


We call such functions "extended real" in order to distinguish them from general complex functions.

## 2.1 The Squire-Trapp method

The method of Squire and Trapp [12, 13] is, in principle, a finite-difference method. It is based on the well known definition of the first-order derivative,


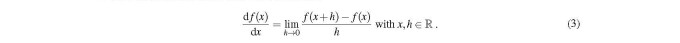


In contrast to the classical finite-element methods, however, the function argument is now incremented along the imaginary axis,


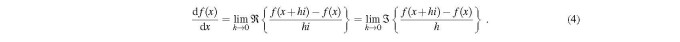


The ℜ and ℐ operators return the real or imaginary, respectively, part of the complex function value. Now ℜ{ *f* (*x*)} is zero by definition for extended-real functions. The differentiation formula thus becomes


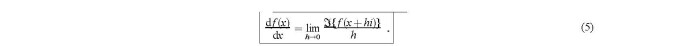


As no difference needs to be computed anymore, it is possible to use very small increments without any loss of precision, e.g., *h* ≈ 10^-100^.

## 2.2 Definition of multicomplex numbers

The extension of the Squire-Trapp method to higher-order derivatives requires a generalization of complex numbers to so-called multicomplex numbers. Recent explorations of multicomplex algebra cover some of the relevalant numerical considerations expanded upon here [14, 15]

A complex number can be written as

*z* = *r*_0_ + *ir*_1_, (6)

where *r*_0_ and *r*_1_ are real numbers and *i* ≡ *i*^(1)^ the imaginary unit. The set of complex numbers can be represented by a plane spanned by a real and an imaginary axis.

For bicomplex numbers a third axis is added, which is characterized by its own imaginary unit, *i*^(2)^. Just as a complex number comprises two real numbers, a bicomplex number *b* comprises two complex numbers *z*_0_ and *z*_1_

*b* = *z*_0_ + *i*^(2)^*z*_1_

 = *r*__00__ + *i*^(1)^r__01__ + *i*^(2)^*r*__10__ + *i*^(1)^*i*^(2)^*r*__11__ . (7)

Similarly, a triplex number comprises two bicomplex numbers, and so on.

The general recursive definition of a multicomplex level *l* is


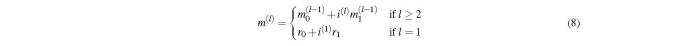


Thus a multicomplex number of level *l* = 1 corresponds to a complex number. Level *l* = 0 is not allowed. The imaginary units of all levels obey the rule


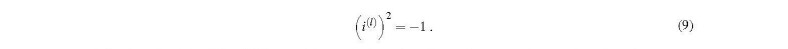


Taken together, Eqs. (8) and (9) permit the evaluation of multicomplex expressions and functions by recursively referring them to complex or real arithmetics. This is explained in more detail in Sec. 6.

A multicomplex number of level *l* can be regarded as a binary tree, where the leaves are the real components. Evidently, there are 2*^l^* real components *r_k_* with *k* = 0,..., 2*^l^* - 1. The binary representation of the subscript *k* may be regarded as "coordinate" of a real component in the tree, for example,

.
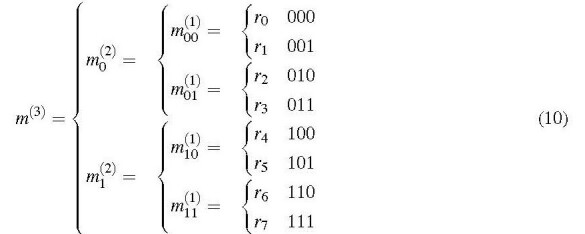


For better clarity, we introduce a "component operator" ${ℭ}_{k}\{\}$ according to


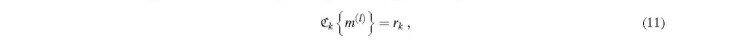


i.e., this operator returns the *k*th real component of a multicomplex number. The binary representation of *k* indicates the sequence of real/imaginary forks taken when traversing the tree. Component ${ℭ}_{0}\left\{m^{\left(l\right)}\right\}=r_{0}$is found in this tree by always taking the real fork; it is the "most real" component. Conversely, always taking the imaginary fork leads to the "most imaginary" component, ${ℭ}_{2^{l}-1}\left\{m^{\left(l\right)}\right\}=r^{2_{l}-1.}$^.^

For a multicomplex number of level 1 (i.e., a complex number), ${ℭ}_{0}$ {} corresponds to the ℜ operator, and ${ℭ}_{1}$ {} corresponds to the ℜ operator.

## 2.3 Multicomplex differentiation-functions of a scalar

Let *f* (*x*) denote a real function of the real variable *x*. It is assumed that *f* can be differentiated at least *l* times with respect to *x*.

As a generalization of the Squire-Trapp method, we now extend *x* to a multicomponent number of level *l*. Consequently, *f* (*x*) also becomes multicomplex. We now consider the following Taylor series:


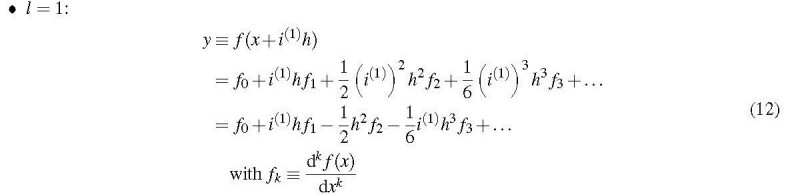


Collecting the real and imaginary terms then leads to


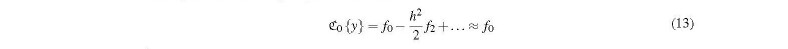


and


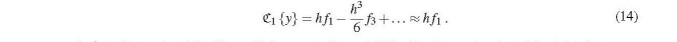


As *h* can be made arbitrarily small, these equations yield the function value *f*_0_ and the 1st-order derivative *f*_1_ practically exactly. Eq. (14) is equivalent to the Squire-Trapp formula, Eq. (5).


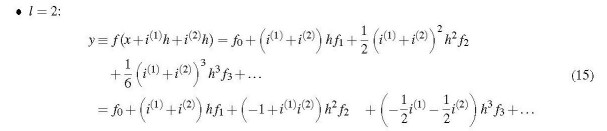


The components of this multicomplex number are (omitting negligible *h*^2^ terms)


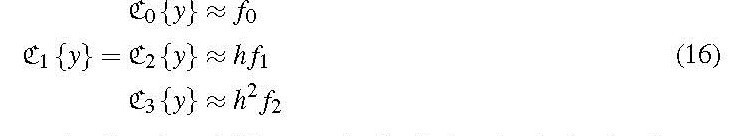


Evidently the evaluation of this multicomplex function yields not only the 2nd-order derivative, but also the 1st-order derivative and the function value.

These results can be generalized for arbitrary levels *l*: The *l*th-order derivative of a function is obtained

as


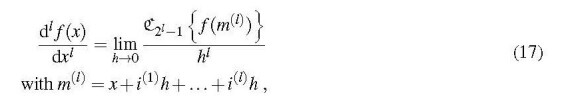


where *h* is a small increment. A proper choice is discussed in Section 3.1. For the practical evaluation the multicomplex argument of *f* can be set up as follows,


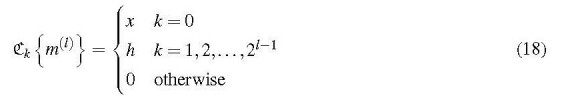


Eq. (17) is the generalization of the Squire-Trapp method for higher-order derivatives. It should be noted that the last, "most imaginary", component of *f* (*m*^(^*^l^*^)^) contains the highest-order derivative. The other derivatives can be extracted from other components of *f* (*m*^(^*^l^*^)^), too. The general formula for the calculation of derivatives of a function of a scalar is


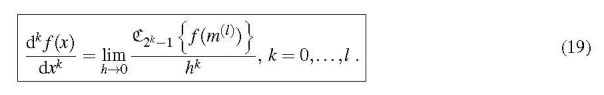


## 2.4 Multicomplex differentiation - functions of a vector

A very attractive feature of multicomplex differentiation is its applicability to functions of more than one variable. Thus it is possible to obtain the derivatives of a function of a vector ***x*** ≡ (*x*_1_, . . ., *x_n_*), including mixed derivatives, according to


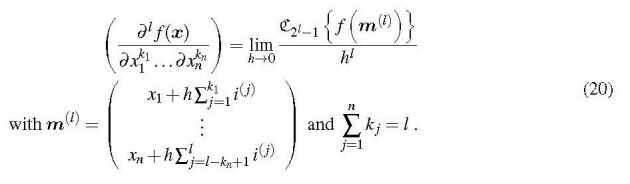


Each component of the multicomplex argument vector *m*^(^*^l^*^)^ receives as many increment terms as the order of the differentiation, *k_i_*, prescribes. To obtain, for example, the mixed third derivative $\left(\partial^{3}f(x)/(\partial x_{1}^{2}\partial x_{2})\right)$it is necessary to evaluate


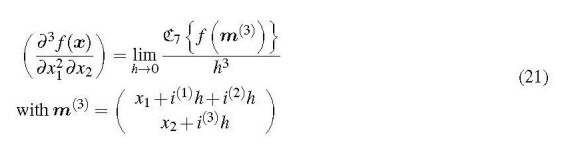


Whether an *i*^(^ *^j^*^)^*h* increment is added to *x*_1_ or to *x*_2_ does not matter, as long as *x*_1_ gets two increments and *x*_2_ gets one.

Just as with the differentiation of functions of scalars, the multicomplex function result contains all lower-order derivatives, too. Their extraction depends on the association of the imaginary units *i*^(^ *^j^*^)^ with the argument components *x_i_*. For the association scheme employed in Eq. (20), the formula for the determination of a derivative having the differentiation orders

$k_{1}^{՚},...k_{n}^{՚}k_{n}$is 


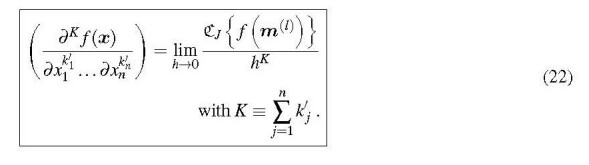


The index *J* is given by


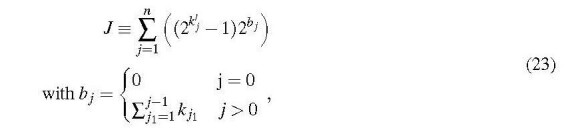


where the *b _j_* are the accumulated orders of differentiation that were used to set up ***m***^(^*^l^*^)^. This expression for *J* may look complicated, but its implementation in a computer program is not, as shown in Section 3.3. For the example given above, Eq. (21), the *b* exponents are *b*_1_ = 0 and *b*_2_ = 2. Then the mixed 2nd-order derivative$\left(k_{1}^{՚}=k_{2}^{՚}=1\right)$ is


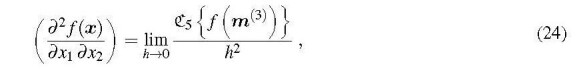


and the pure 2nd-order derivative with respect to *x*_1_ $\left(k_{1}^{՚}=2,k_{2}^{՚}=0\right)$is


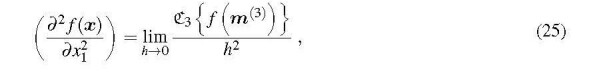


These two (and more) derivatives are obtained without additional computational costs when

$\left(\partial^{3}f(x)/(\partial x_{1}^{2}\partial x_{2})\right)$ is computed.

## 3 Application

## 3.1 The differentiation increment

It is a pleasant feature of multicomplex differentiation that there is no need to provide a problem-specific control parameter. It is necessary to choose a differentiation increment *h*, but this should simply be made as small as possible. This is in contrast to real finite-difference methods, where the determination of an optimal step width that balances the round-off errors and the termination errors is a nontrivial task. Complex-integration methods, on the other hand, require an integration radius, and an improper choice either spoils the accuracy or slows the calculation down[16].

However, the proposition to make *h* as small as possible needs some further explanations. Higham proposes *h* = 10^-100^ for the Squire-Trapp method, but this value cannot be adopted for multicomplex differentiation without further consideration. The formulas for *l*th-order derivatives, Eqs. (17) and (20), contain the term *h^l^*. Now standard double-precision arithmetics (IEEE 754-2019 [17]) imposes a lower boundary for this power, approximately *h^l^* > 10^-308^. It is therefore advisable to let *h* depend on *l*. We found that *h* ≈ 2^-664/^*^l^* works well^1^.

There is, however, a problem of which the users of multicomplex differentiation should be aware: The differentiation formulas Eqs. (17) and (20) rely on the fact that *O*(*h*^2^) terms from the underlying Taylor expansions can be neglected. This leads to the approximate restriction *h*^2^ < *Ɛ*, where *Ɛ* ≈ 10^-15^ is the relative precision of double-precision arithmetics. This limits the order of differentiation to about 12 (when using the formula for *h* given above).

## 3.2 Numerical effort

Another consideration is that a multicomplex number of level *l* comprises 2*^l^* real components (e.g., 1024 components for a 10th-order derivative!). One can therefore expect the storage requirements as well as the computing time to increase exponentially with the order of differentiation.

In contrast to this, the numerical effort of differentiation by complex integration increases approximately linearly with *l*, so that such methods would probably be preferable for higher-order differentiations.

Fortunately, most problems in fluid thermodynamics require 1st- and 2nd-order derivatives only, e.g., the computation of entropy, enthalpy, chemical potential or compressibility from a fundamental equation (Helmholtz energy equation). The calculation of critical curves of mixtures (including stability analysis) requires up to 4th-order derivatives. At present, 5th- and higher-order derivatives appear in calculations of tricritical states or higher-order critical states of similar complexity only, which are needed for the construction of global phase diagrams [18-20]. All these applications are therefore within the useful range of multicomplex differentiation.

## 3.3 Implementation hints

Multicomplex arithmetics is not (yet) routinely included in most modern computer languages. We therefore make multicomplex libraries for C++ and Python available [21]. Furthermore, we describe basic multicomplex operations as well as multicomplex versions of some frequently used functions in Sec. 6.

Section 7 offers some commented C++ code for multicomplex differentiation. In this section we demonstrate the application of multicomplex differentiation to some thermodynamic problems.

In our C++ implementation, multicomplex numbers are instances of a multicomplex class. The ℭ {} operator, which sets or retrieves the real components of a multicomplex number, is implemented as a member function of that class:


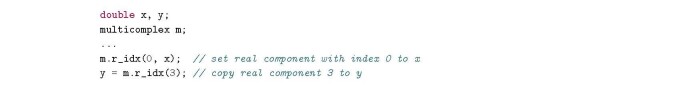


For our application examples we define the dimensionless residual Helmholtz energy function of a pure fluid as a function of the molar density ρ and the temperature *T* ; for simplicity we use the model of van der Waals here,

where *a* and *b* are substance-specific parameters; of course more realistic (and more complicated) Helmholtz energy functions can be used instead. We implement the van der Waals Helmholtz energy function in C++ as 


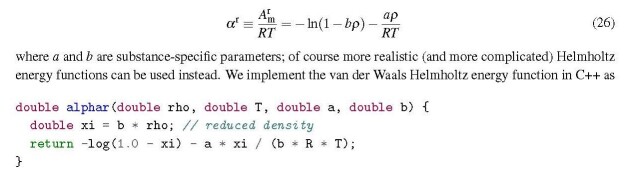


In order to create a multicomplex version of this function, we merely have to add a template declaration:


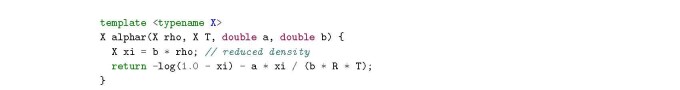


## 3.3.1 The pressure of a pure fluid

The pressure is obtained as


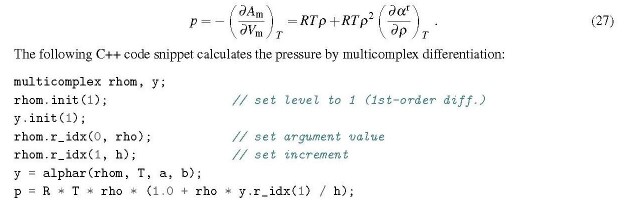


## 3.3.2 Virial coefficients of a pure fluid

The (density-dependent) virial series is


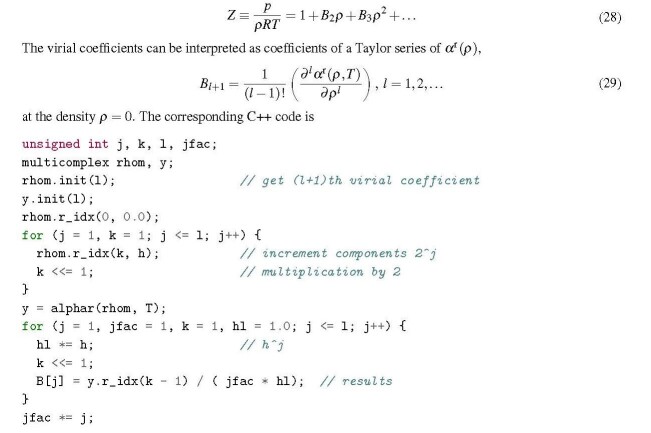


It should be noted that a single evaluation of the multicomplex Helmholtz energy function generates all virial coefficients up to *B_l_*_+1_ at once.

## 3.3.3 The isochoric pressure coefficient

This coefficient is the temperature derivative of the pressure, and therefore a mixed derivative of *α*^r^(*ρ*, *T*),


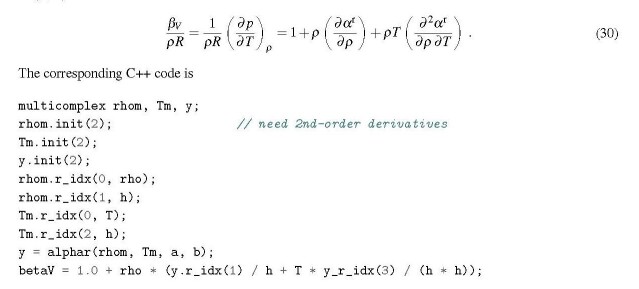


In this case the highest orders of differentiation with respect to *ρ* and *T* are both 1 (*k*_1_ = *k*_2_ = 1, *l* = 2). The *b _j_* coefficients (Eq. (23)) are 0 and 1, and therefore (*∂α*^r^/*∂ρ*) (with $k_{1}^{՚}=1,k_{2}^{՚}=2$) is found at the index *J* = 1.

It should be noted that again merely one multicomplex function call is needed to obtain both partial derivatives.

## 3.3.4 Functions of a vector-general implementation

A general recipe for setting up the multicomplex arguments for a differentiation of a function an *n*-dimensional argument, *f* (***x***), with the orders *k*_1_, *k*_2_, . . ., *k_n_* is:


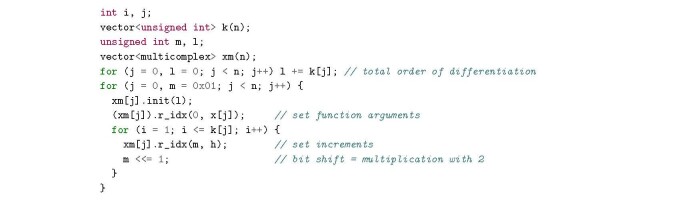


This code snippet follows the C/C++ array index convention-i.e., arrays start with index 0-and assumes that a convenient array class has been defined. Here the class std::vector is used, but there are several alternative classes.

The next step is the evaluation of the multicomplex function:


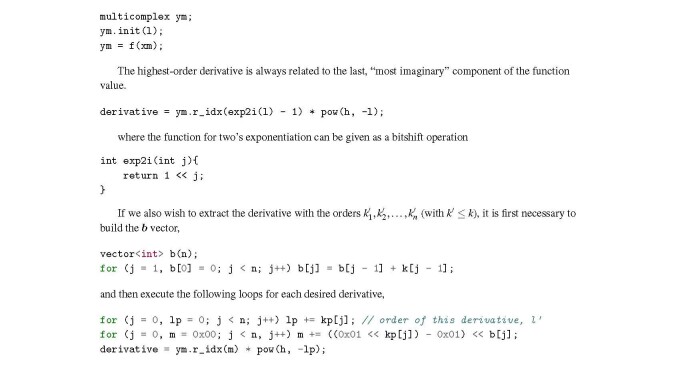


## 4 Results

In this section we time some calculations with the multicomplex approach. Benchmark results in C++ from the library are included in Table 1 for some common mathematical functions. Aside from the ln(x) function (and the pow function which implicitly calls the ln(x) function), for which a few microseconds are required to evaluate the first derivative, the first derivatives of all other mathematical functions are evaluated in less than a microsecond. To put that in perspective, there is on the order of 1 *μ*s overhead to make a call from Python to C++, so the computational penalty is not too severe. Other approaches, most notably automatic differentiation approaches, have much lower computational overhead, but introduce some new challenges from an implementation standpoint. The benchmark script is available in the source code of multicomplex.

**Fig. 1:**
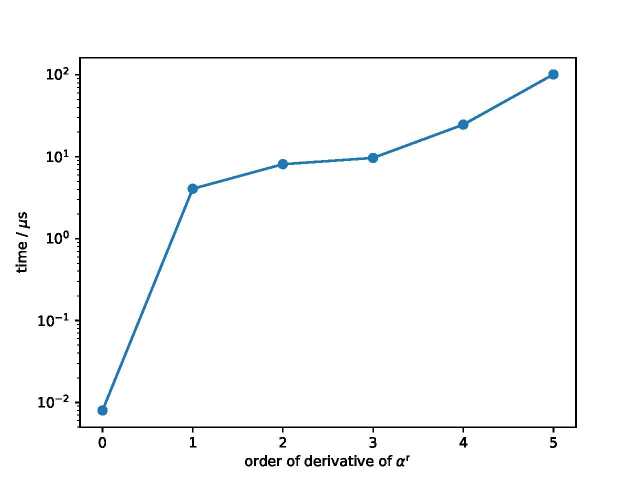
Timing of the density derivatives ${\left(\frac{\partial^{n}(\alpha^{r})}{\partial \rho^{n}}\right)}_{T}$of the van der Waals equation of state in C++.

**Table 1:** Timing results for some common mathematical expressions. The test name matches the same field in the C++ code of the bench.cxx file of multicomplex. Tests were run on an Intel i7-10810U CPU running windows 10 with the benchmarking tools of the Catch2 package, and the XML output was processed to generate the table.

test	function / ns	first derivative / ns
time x^3	1.0	458.0
time x^3, but with pow(x,int)	42.0	671.0
time x^3, but with pow(x,double)	40.0	6676.0
time 1/x	1.0	1066.0
time sin(x)	13.0	452.0
time cosh(x)	16.0	555.0
time exp(x)	9.0	448.0
time ln(x)	7.0	5878.0
time cos(x)*sin(x)	20.0	659.0

To come back to the van der Waals equation of state, we provide timing of the density derivatives of the equation of state in Fig. 1, in C++, as a function of the number of derivatives desired. We should caution that this is a somewhat unfair comparison because the evaluation of the EOS itself requires only a few computational cycles as it invokes a logarithm but otherwise only the negation, subtraction, multiplication, and division operators, each of which can be very efficiently carried out in double precision. The jump from the function evaluated in double precision to first derivative is rather steep, but after that, higher derivatives do not introduce much additional computational penalty.

## 5 Conclusion

Numerical differentiation with the multicomplex finite-step method is a very versatile and easy-to-use tool for calculating derivatives of functions with nearly machine precision. In contrast to the Squire-Trapp method, of which the multicomplex step method is an extension, it is applicable to higher-order derivatives. In contrast to methods based on complex integration, the multicomplex step method can obtain mixed derivatives without introducing computational complications like integration in more than one dimension. Moreover, the multicomplex method can obtain several derivatives of a function at the same time without additional computing effort.

Another pleasant feature of the new method is that there is no control parameter that needs to be determined or optimized, like the differentiation increment of real finite-step methods (e.g., the methods of Romberg or Ridder) or the integration radius of the complex-integration methods. The multicomplex step method does use an increment, but its value can be chosen within several orders of magnitude; choosing the increment size is uncritical and can be safely left to the algorithm.

There are, however, some limitations: The attainable size of the increment on a computer using double-precision arithmetics as well as CPU time considerations restrict the useful range of the order of differentiation to approximately 12. But this is more than enough for most thermodynamical applications-calculation of phase equilibria or caloric properties from fundamental equations (Helmholtz energy as a function of density, temperature, and composition) or of critical states.

A practical disadvantage is, at present, that the contemporary computer languages cannot handle multicomplex numbers. We therefore describe the implementation of multicomplex calculations-arithmetic operations as well as some important functions-in Sec. 6. Ready-to-use program libraries for C++ and Python can be found in Ref. [21]; the C++ library can also be invoked from Fortran programs. Furthermore, we provide Python sample code in Section 9.

## 6 Appendix A: Multicomplex arithmetics and functions

As multicomplex arithmetics is not widely known, yet, and not included in standard compiler libraries, we present here some rules for the manipulation of multicomplex numbers as well as methods for the evaluation of some frequently needed functions.

As will be explained below, some well-behaved real functions have got rather problematic complex or multicomplex counterparts. But even such functions can be evaluated reliably for real-dominant arguments. Fortunately, this is exactly the case for multicomplex differentiation.

The differentiation recipes Eqs. (19), (20), and (22) work reliably if these conditions are fulfilled:

1. If the multicomplex argument *m*^(^*^l^*^)^ is effectively real (i.e., all${ℭ}_{k\neq 0}\left\{m^{\left(l\right)}\right\}=0),$ then *f* (*m*^(^*^l^*^)^) must be real, too.

2. If the multicomplex argument is real-dominant (i.e. all${ℭ}_{k!=0}\left\{m^{\left(l\right)}\right\}=\text{O}(h)),$ then *f* (*m*^(^*^l^*^)^) must be real-dominant, too.

3. The differentiation increment *h* must be small.

Python and C++ code for the implementation of multicomplex arithmetics can be found here [21]. The C++ library can also be invoked from Fortran programs.

### 6.1 Arithmetic operations

Let *a*^(^*^l^*^)^ and *b*(*l*) denote two multicomplex numbers of level *l*. Then their sum is obtained as

Unless the divisor is zero in the last case, all these operations can be applied to arbitrary multicomplex numbers and are therefore safe to use.

Addition of and multiplication by real numbers are described here:

For level *l* = 1 the well-known rules for complex arithmetics can be used.

Finally it is useful to define a "promotion rule": If an arithmetic operation or a value assignment is performed with multicomplex numbers of different levels, the number with the lower level gets promoted,

### 6.2 Comparison operations

Two multicomplex numbers are equal if all their real components are equal, and unequal otherwise. The recursive formulation of this rule is

As already for complex numbers, the comparison operators < and > cannot be defined. It is, however, possible to define the norm of a multicomplex number, which is the square root of the sum of its real components,

### 6.3 Exponential and trigonometric functions

These functions can be implemented as follows [22]:

The disadvantage of this exp() implementation is that a large negative argument, e.g., *e*^-1000^, may cause an overflow error in the hyperbolic functions sinh and cosh called by sin and cos. Instead, it is better to implement the exponential function as

### 6.4 The natural logarithm function

The natural logarithm of a complex number *z* = $re^{i\phi }$ is given by

This is evidently a multivalued relation. The complex log() function of the C/C++ standard library returns the principal value of ln(*x*), namely the value for *k* = 0.

The test relation

is not guaranteed to work, because-depending on the value of *k*-the sine and cosine terms will change their signs.

The ambiguity of evaluations of the ln() function is even aggravated in multicomplex algebra. A general implementation of this function in multicomplex algebra is therefore difficult, perhaps even impossible.

Fortunately, the multicomplex numbers appearing in the course of numerical differentiation are of the real-dominant type. For such numbers an efficient and reliable iterative scheme can be given. We set *y* = ln *x* and apply Halley's method,

This iteration can be carried out with multicomplex numbers. It does not require problematic steps. Halley's method has a convergence order of 3, which means that, in the vicinity of the solution, the number of valid digits triples with each step. If the iteration is started at *y*_0_ = ln(${ℭ}_{0}${*x*}), convergence is usually achieved after 1-2 steps.

### 6.5 ln(1 + *x*)

This function can be conveniently evaluated by recursion,

### 6.6 The arctangent function

Like the logarithm function, the arctangent function is safe for real-dominant multicomplex arguments only. It can be calculated iteratively by means of Newton's method,

should be used. For the purpose of numerical differentiation, the iteration should be started at *y* = ℭ_0_ {*x*}.

### 6.7 The root function

Iteration can also be used to obtain roots or multicomplex numbers. $y=\sqrt[{n}]{c}$Substituting *f* (*y*) = *y^n^* - *x *into Newton's scheme yields the iteration formula

### 6.8 The power function

Integer powers, *x^n^*, should be handled by multiplication. Arbitrary real powers can be obtained from

## 7 Appendix B: Commented C++ subroutines for multicomplex differentiation

The following samples demonstrate the layout of some multicomplex differentiation subroutines. We do not list all required C++ headers (*.h files) here, as these are rather user-specific.

### 7.1 The increment function

The following function computes an increment for an *l*th order multicomplex differentiation.

### 7.2 Differentiation of a function of a scalar

The next function computes the *l*th-order derivative of a multicomplex (extended-real!) function *f* (*x*) at the location *x*.

The following version of the subroutine returns more than one derivative. The derivatives are specified by means of an "order vector". If for example, the first and the second-order derivatives are needed, the order vector is a two-component vector defined as $\vec{o}$ = (1, 2). We assume that an appropriate vector class has been defined.

The result is a vector containing the derivatives as specified by the order vector.

### Differentiation of a function of a vector

The following example computes a derivative of a multicomplex function *f* (***x***), where ***x*** is an *n*-dimensional vector. The derivative is specified by means of the "order vector" *o*, which contains the order of differentiation for each dimension. For example, the second-order mixed derivative of a function of a two-dimensional vector, (*∂* ^2^ *f* (***x***)/*∂x*_0_ *∂x*_1_), is specified with ***o*** = (1, 1).

## 8 Appendix C: Differentiation by complex integration

### 8.1 Function of one variable

Complex integration methods are based on Cauchy's integral theorem, which relates the value of a function *f* (*z*) or any of its derivatives at the location *z*_0_ to a path integral,

The integration can accomplished numerically by means of trapezoidal integration . This is usually known as a rather inferior method, but for periodic functions it is very efficient: With increasing number of evaluation nodes *N*, the error of the integral decreases faster than any power of 2*π*/*N*.

The advantage of using an *integral* formula to obtain derivatives is that numerical integration-quite in contrast to numerical differentiation by means of finite-difference formulas-does not suffer from cancellation errors. If the radius of the integration path is chosen large enough (so large that *f* (*z* + *z*_0_) shows a significant variation), derivatives can be computed practically with machine precision.

An efficient application of Cauchy's theorem to extended-real functions is the method of Lyness and Moler [10]. It constructs a series of approximations *y_m_* for the *n*th-order derivative,

Convergence is usually quite rapid; the calculation can safely be terminated when the difference of two successive approximations falls below a predefined threshold.

### 8.2 Functions of more than one variable

A straightforward extension of Eqs. (60) or (61) to functions of two or more arguments is possible, but complicated and computationally expensive, as it requires integrations in more than one dimension. This makes it difficult to compute mixed derivatives. Fortunately there is a workaround by means of the formalism of directional derivatives.

The directional derivative of a function of an *N*-dimensional vector argument, *f* (***x***) is defined by [23]

The three second-order derivatives can be computed with Eq. (61), using *ξ* as (single) argument with three different ***u*** vectors. It should be noted that Eq. (70) is not a finite-difference differentiation formula and therefore does not necessarily suffer from cancellation errors.

## 9 Appendix D: Python example

This example in the python programming language (version 3.8) with the van der Waals EOS uses the

multicomplex Python package, version 0.12
